# Do empowered women receive better quality antenatal care in Pakistan? An analysis of demographic and health survey data

**DOI:** 10.1371/journal.pone.0262323

**Published:** 2022-01-06

**Authors:** Muhammad Asim, Waqas Hameed, Sarah Saleem

**Affiliations:** Department of Community Health Sciences, Aga Khan University, Karachi, Pakistan; Marie Stopes International, PAKISTAN

## Abstract

**Introduction:**

Quality antenatal care is a window of opportunity for improving maternal and neonatal outcomes. Numerous studies have shown a positive effect of women empowerment on improved coverage of maternal and reproductive health services, including antenatal care (ANC). However, there is scarce evidence on the association between women’s empowerment and improved ANC services both in terms of coverage and quality. Addressing this gap, this paper examines the relationship between multi-dimensional measures of women empowerment on utilization of quality ANC (service coverage and consultation) in Pakistan.

**Methods:**

We used Pakistan Demographic and Health Survey 2017–18 (PDHS) data which comprises of 6,602 currently married women aged between 15–49 years who had a live birth in the past five years preceding the survey. Our exposure variables were three-dimensional measures of women empowerment (social independence, decision making, and attitude towards domestic violence), and our outcome variables were quality of antenatal coverage [i.e. a composite binary measure based on skilled ANC (trained professional), timeliness (1^st^ ANC visit during first trimester), sufficiency of ANC visits (4 or more)] and quality of ANC consultation (i.e. receiving at least 7 or more essential antenatal components out of 8). Data were analysed in Stata 16.0 software. Descriptive statistics were used to describe sample characteristics and binary logistic regression was employed to assess the association between empowerment and quality of antenatal care.

**Results:**

We found that 41.4% of the women received quality ANC coverage and 30.6% received quality ANC consultations during pregnancy. After controlling for a number of socio-economic and demographic factors, all three measures of women’s empowerment independently showed a positive relationship with both outcomes. Women with high autonomy (i.e. strongly opposed the notion of violence) in the domain of attitude to violence are 1.66 (95% CI 1.30–2.10) and 1.45 (95% CI 1.19–1.75) and times more likely to receive antenatal coverage and quality ANC consultations respectively, compared with women who ranked low on attitude to violence. Women who enjoy high social independence had 1.87 (95% CI 1.44–2.43) and 2.78 (95% CI 2.04–3.79) higher odds of quality antenatal coverage and consultations respectively, as compared with their counterparts. Similarly, women who had high autonomy in household decision making 1.98 (95% CI 1.60–2.44) and 1.56 (95% CI 2.17–1.91) were more likely to receive quality antenatal coverage and consultation respectively, as compared to women who possess low autonomy in household decision making.

**Conclusion:**

The quality of ANC coverage and consultation with service provider is considerably low in Pakistan. Women’s empowerment related to social independence, gendered beliefs about violence, and decision-making have an independent positive association with the utilisation of quality antenatal care. Thus, efforts directed towards empowering women could be an effective strategy to improve utilisation of quality antenatal care in Pakistan.

## Introduction

High-risk pregnancies are more common in low- and middle-income countries (LMICs) and pose greater risk for the survival of newborns as compared with high-income countries [1, 2]. Effective antenatal care can reduce up to 50–70% of maternal and neonatal deaths through the timely detection and prevention of risk factors associated with potential obstetric complications [3]. Although the recommended ANC coverage has substantially increased in the last two decades in LMICs [4], the quality of ANC is still well emphasized in mainstream health systems. In 2017, about 295,000 women died during pregnancy and childbirth, and ninety-four percent of these maternal deaths took place in low- and middle-income countries (LMICs) [5], with almost one third occurring in South Asia. Moreover, between one–third and one–half of these pregnancy–related deaths are due to preventable complications, such as eclampsia and haemorrhage, directly related to inadequate quality of care [5].

In the era of Sustainable Development Goals, there is a global shift in the perspective from crude coverage to effective coverage of ANC. Attending to recommended ANC visits is only a proxy indicator of service provision and health system performance as it ignores the content and quality of antenatal visits [6]. Merely attending ANC visits does not imply that pregnant women will receive all essential components of ANC [7, 8]. Aligning with the SDG 3, the World Health Organization (WHO) has proposed an integrated focused ANC model which emphasizes on quality over quantity of antenatal visits for positive pregnancy experiences and improved birth outcomes [3]. According to the WHO, expecting mothers should get at least eight ANC visits from skilled professionals and these consultations should include obstetrical examination, screening and testing, health education, advice, and, counselling on maternal and neonate care [3] in a respectful manner.

While inequalities in the coverage and utilization of maternal health services are widely reported [9, 10], limited evidence illuminates disparities with a focus on quality of maternal health services [11]. The available evidence shows inequalities in quality of ANC services by wealth, age, education level, and, working status of women [12, 13]. The focus of this study is to fill this gap by examining the relationship between women empowerment and quality of antenatal care. The concept of women empowerment is relatively subjective, complex, and multidimensional, and is fundamentally linked with women’s ability to use resources effectively to achieve desired outcomes [14, 15]. The multidimensional nature of empowerment makes it challenging for the researcher to measure the phenomenon objectively. Several indices have been used in studies in relation to health outcomes, and each has its own limitations. Primarily, they often focus on certain aspects of empowerment do not comprehensively capture the broad concept of women empowerment [16, 17].

We used a survey-based measure of a Women’s emPowERment (SWPER) which has been recently developed and validated based on nationally representative demographic and health survey data from low- and middle-income countries [16]. Based on a conceptual framework proposed by Miedema et al., [17] the global measure of empowerment measures captures three dimensions: social independence (preconditions that enable women to achieve their goals), decision making (extent of women’s participation in household decisions) and attitude to violence (intrinsic agency, as a proxy for women’s incorporation of gender norms-related acceptability of violence).

Women empowerment is an important indicator of maternal health care services utilization. Evidence show a positive effect of empowerment on the use of contraception, access to and use of antenatal care [18], and skilled birth [19, 20]. Specific to quality of ANC care, previous studies both globally and within the South Asian context-specific to Pakistan only conceptualized the uni-dimensional women empowerment (decision-making autonomy or denial spousal violence) with crude coverage of antenatal care instead of quality of ANC [21–23]. This study used the multidimensional variables for women empowerment and created the index of quality of ANC. In view of the limited evidence, the objective of current study is to examine the relationship between multi-dimensional measure of women empowerment and utilization of quality of ANC in Pakistan.

## Methods

### Data

We conducted a secondary data analysis of Pakistan Demographic Health Surveys 2017–18 (PDHS), a most recent publicly available nationally representative dataset. Using a two-stage stratified cluster sampling design, the survey was conducted across four provinces of Pakistan (Punjab, Sindh, Khyber Pakhtunkhwa, and Baluchistan), the Islamabad Capital Territory, Federally Administered Tribal Areas (FATA), Gilgit Baltistan (GB), and Azad Jammu & Kashmir (AJK). At first, 16-urban-rural strata was created from above stated eight regions. Second, 580 clusters were selected with probability proportional to number of households in each cluster. Third, a 28 household per cluster (16,240 in total) were selected from each cluster with an equal probability of selection.

We used women’s dataset which comprised of 15,068 observations on ever-married women aged 15–49 years. GB and AJK regions were excluded because they had a different sampling frame. Further, we restricted our data to women who had a live birth in the past 5 years before the survey. Hence, our analysis is based on 6,602 women. Information about ANC was only collected and analysed for the most recent birth.

### Measures

Our main exposure variable was three-dimensional measures of women’s empowerment, and our outcome variable about was utilization of quality ANC services.

#### Outcome variables

There are two outcome variables that were broadly categorised as: quality of ANC coverage and quality ANC consultation (i.e. utilisation of essential ANC components). The construction of quality antenatal coverage (binary variable) was based on three key indicators such as: skilled antenatal care (antenatal care received from a doctor, nurse, midwife, or lady health visitor), timeliness (first antenatal care was received during the first trimester), and sufficiency (received at least four antenatal care visits). Women who received ANC from skilled health care providers, visited for ANC during the first trimester, and completed at least four antenatal care visits was coded “1” and otherwise ‘0’.

The quality of antenatal consultations was based on receiving of essential components of ANC that included: blood pressure examination, blood examination for iron deficiency anaemia, urine examination, received at least two tetanus toxoid vaccination shots, consumed iron and folic acid tablets/syrup for at least 100 days, received advise on early initiation of breastfeeding, receive advise on exclusive breastfeeding, and receive advised on balanced diet. The essential elements of a focused approach to antenatal care were used by the World Health Organization [6]. Women who received at least seven components of ANC were coded as “1” received less than seven components coded as “0”.

#### Construction of women’s empowerment indices

We used the SWPER scale to construct three domains of women’s empowerment that included: attitude to violence, social independence, and women’s decision making [16]. The SWPER index measure women empowerment that has been validated for global comparisons with the DHS data from 62 low- and middle-income countries. This index uses 14 indicators to create three domains of empowerment such as social independence, decision making, and attitude towards violence.

Attitude to violence was measured by asking whether women justified wife beating under five conditions, such as, if she: burns the food, goes out without telling husband, neglects the children, argues with husband, or refuses to have sex with husband. The responses are categorized as “beating justified” or “not” in each five domains (justified = –1; don’t know = 0; not justified = 1). The measure of social independence was constructed based on five questions which included: age at first marriage and age at first birth as continuous variables, frequency of reading newspaper or magazine, absolute difference between women and spousal education and age. Age and schooling were coded as wife’s assets minus husband’s assets, with positive values indicating women’s higher asset attainment relative to her husband. For the measure of decision making, women were asked who typically decide on: respondent’s health care, large household purchases, and visiting family or relatives. These measures were categorized into low, medium and high categories in accordance with the standard cut-off provided for the South Asian region [16].

#### Covariates

Women’s age, place of residence, wealth index, and number of children were kept as covariates in this study. The age of respondents was divided into three categories such as <20 years, 21–34 years and 35–49 years. Place of residence was categorized as either rural or urban. The economic status of women was factored in the form of wealth quintile, a measure generated through principal component analysis technique. The variable of total number of pregnancies was categorized as 1–2, 3–4 and 5+ pregnancies.

### Data analysis

**Fig 1:**
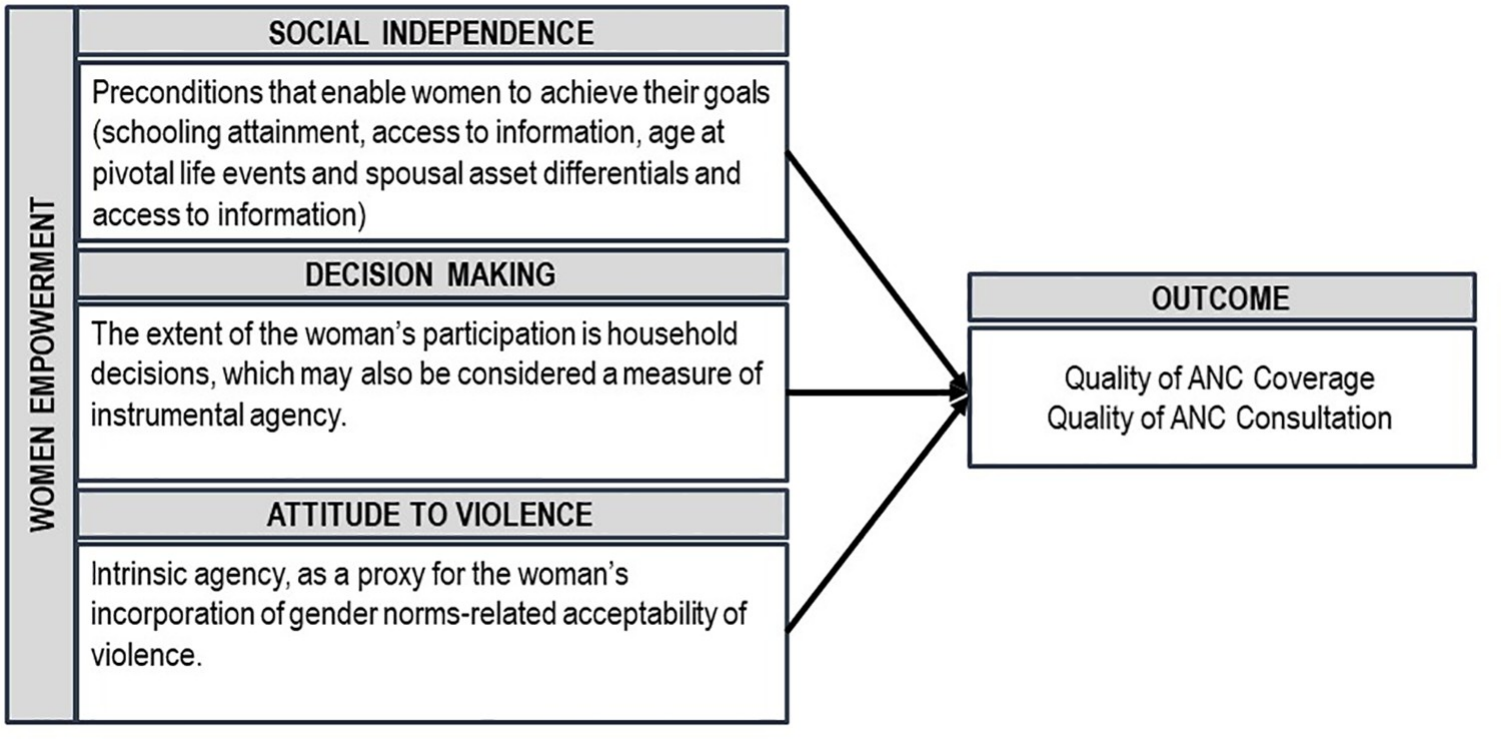
Analytical framework of women’s empowerment.

The data were analysed through Stata version 16.0 software by adjusting for the complex survey design, strata, clusters, and probability sampling using individual weights. Descriptive analysis was used to examine the socio-demographic characteristics, level of empowerment, antenatal coverage, and components of antenatal care received during visits. Pearson Chi-square test was used to test the crude association between women’s empowerment and utilisation of ANC. Finally, multiple logistic regression was used to assess the effect of women empowerment and ANC outcomes, after adjusting for the covariates described above. The results of multiple logistic regression are presented in the form of odds ratios (ORs) and adjusted odds ratios (aORs) with 95%CIs.P-value of <0.05 was considered significant. The analysis of this study is guided by the framework presented in Fig 1.

### Ethical approval

We used publicly available secondary data from PDHS 2017–18. Hence, ethical approval was not required. However, the survey protocol was reviewed and approved by the National Bioethics Committee, Pakistan Health Research Council, and ICF Institutional Review Board [24].

## Results

The socio-demographic characteristics of 6,602 women aged 15–49 years who had a live birth in the past years are presented in Table 1. Almost half of women (47.8%) had no formal education and only 13.6% women had more than 9^th^ grade education. Most of the women resided in the rural areas (66.5%). About three in five women were between 20–34 years, and 34.6% were under the age of 20 years. Most of the women had 1–2 children (40.8%), 33.6% had 3–4 children and 26.6% had 5 or more children.

**Table 1:** Socio-demographic characteristics of study sample.

Characteristics	(n = 6602) %
**Age at birth**	
<20	34.6
20–34	59.8
35–49	5.6
**Women education**	
No education	47.8
Primary (up to 5 grade)	16.4
Secondary (up to 8 grade)	22.2
Higher (9 grade or above)	13.6
**Parity**	
1–2	40.8
3–4	32.6
5+	26.6
**Place of residence**	
Rural	33.5
Urban	66.5

**Fig 2:**
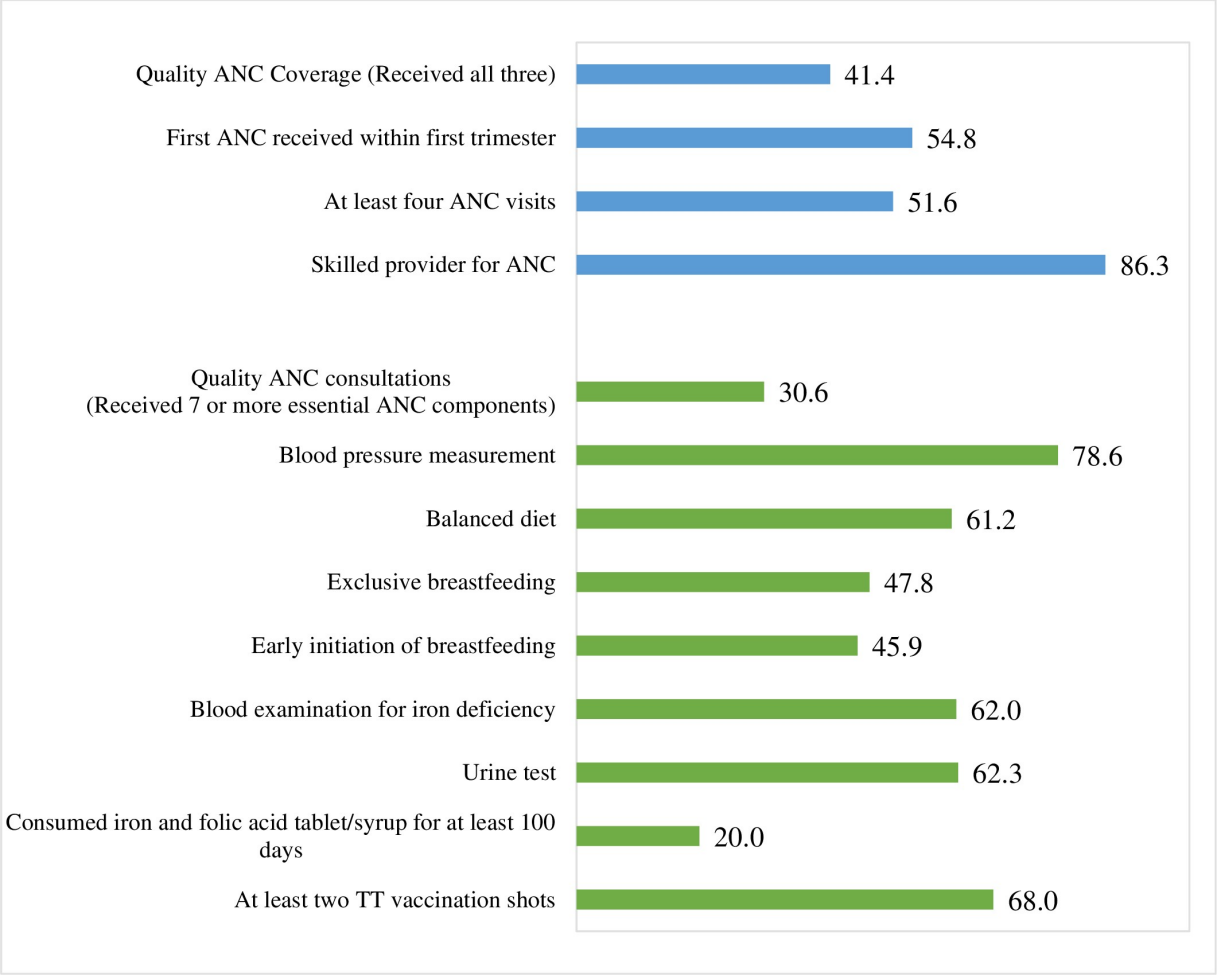
Quality of ANC coverage and consultation during last pregnancy.

Overall 41.4% of women reported to have received quality ANC service coverage–that is, made their first interaction with a healthcare provider during first trimester, made at least four ANC visits, and received ANC from a skilled service provider. About 30% of the women received quality ANC consultation i.e. received at least seven essential component of ANC out of eight. With respect to each ANC component, approximately 4 in every 5 women had their blood pressure measured and 68% received at least two TT vaccine shots. On the contrary, consumption of iron and folic acid tablet/syrup for at least 100 days was reported by only 20% of the women (Fig 2).

**Fig 3:**
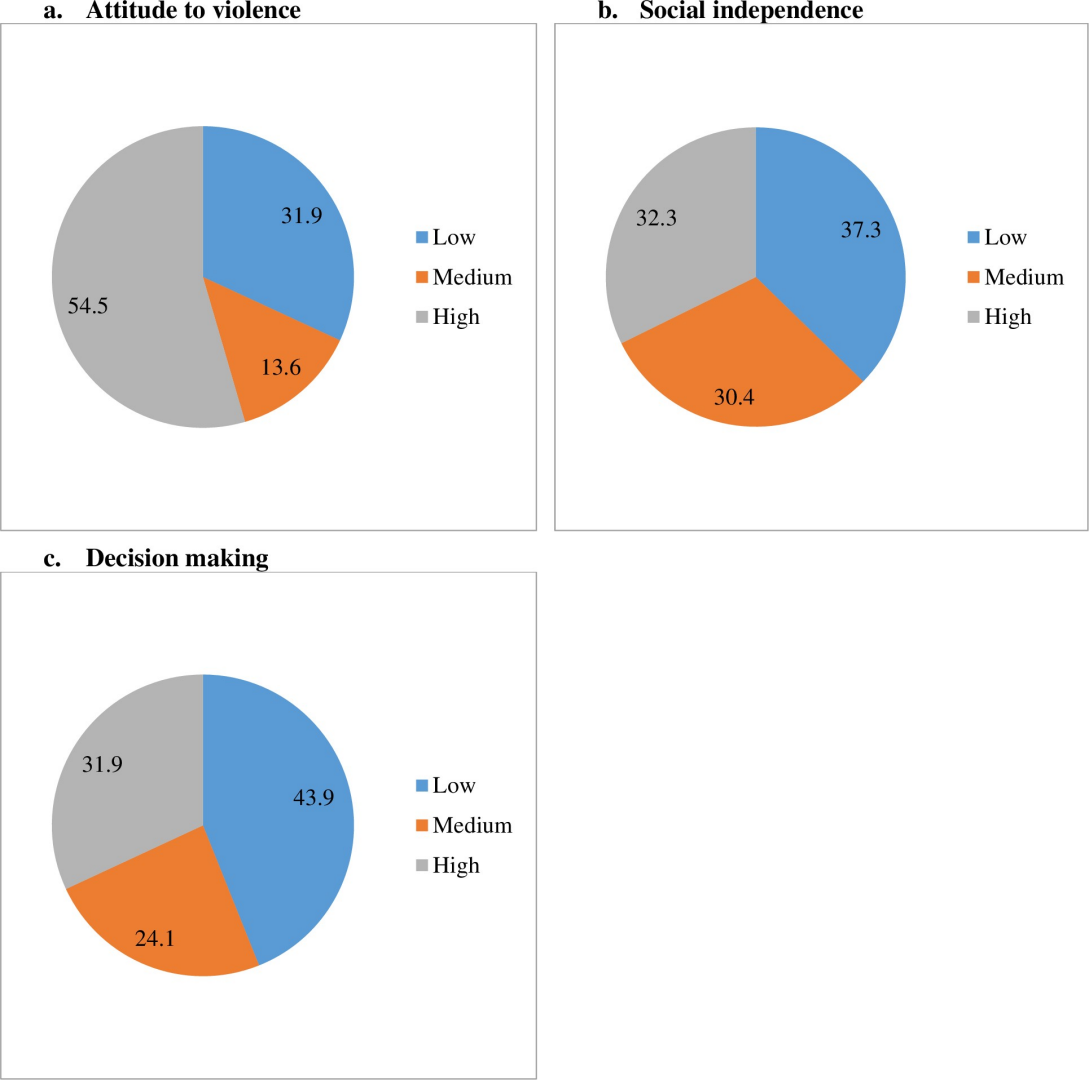
a–c: Level of women empowerment according to different dimensions.

Women’s levels of empowerment according to four different dimensions are presented in Fig 3A–3C. More than half (54.5%) of the women completely rejected domestic violence. However, one-third (31.9%) had accepted domestic violence in certain contexts. A little less than half, (43.6%) of the women had low decision-making autonomy in the household. Women were found to be approximately equally distributed into low, medium and high empowerment on the index of social independence. Three-fourth of the women reported to have no household autonomy for decision making in terms of accessing health care.

Table 2 shows the crude and adjusted relations between measure of women empowerment and ANC service coverage. The crude odds ratios (ORs) showed a clear and consistent positive effect of all measures of women empowerment on ANC service coverage. Although, the magnitude of relationship (ORs) decreased after adjustment of potential covariates but remained significant for all three measures. Compared with women who ranked low in attitude to violence (i.e. least strongly opposed the notion of violence), the odds of ANC service coverage was 1.37 and 1.45 times higher among those who were ranked in medium and high category (i.e. strongly opposed the notion of violence), respectively. Similarly, women having medium (aOR = 1.51) or high (aOR = 1.87) level of social independence had higher ANC coverage as compared to women with low social independence. The odds of ANC coverage also increased with the level of women’s autonomy in household decision making.

**Table 2:** Unadjusted odds ratios (UOR), adjusted odds ratios (AOR), and 95% confidence intervals (CI) of quality antenatal coverage.

Women empowerment	Unadjusted		Adjusted^1^	
	OR	95% CI	AOR	95%CI
Attitude to violence				
Low (least strongly opposed violence)	1		1	
Medium	1.85	1.46–2.35	1.37	1.07–1.77
High (strongly opposed violence)	2.95	2.47–3.52	1.45	1.19–1.75
Social independence				
Low	1		1	
Medium	2.14	1.81–2.52	1.51	1.22–1.88
High	4.27	3.53–5.17	1.87	1.44–2.43
Decision making				
Low	1		1	
Medium	1.48	1.25–1.76	1.32	1.09–1.58
High	1.66	1.40–1.99	1.56	1.27–1.91

^1^Adjusted for women age, education, number of children, place of residence and wealth quintile.

The relationship between women empowerment and quality ANC consultation is presented in Table 3. As in the case of ANC coverage, we found a similar effect of women empowerment on quality of ANC consultations. The crude ORs depicts a significant positive effect of all dimensions of empowerment on ANC consultation (i.e. uptake of seven essential ANC components out of eight). The associations remained significant for all measures of women empowerment in the adjusted model.

**Table 3:** Unadjusted odds ratios (UOR), adjusted odds ratios (AOR), and 95% confidence intervals (CI) of quality ANC consultation.

Women empowerment	Unadjusted		Adjusted^1^	
	OR	95% CI	AOR	95%CI
Attitude to violence				
Low (least strongly opposed violence)	1		1	
Medium	1.56	1.21–2.02	1.10	0.85–1.44
High (strongly opposed violence)	3.35	2.66–4.21	1.66	1.30–2.10
Social independence				
Low	1		1	
Medium	2.24	1.76–2.85	1.66	1.27–2.18
High	5.38	4.26–6.80	2.78	2.04–3.79
Decision making				
Low	1		1	
Medium	1.54	1.25–1.91	1.34	1.07–1.66
High	2.1	1.71–2.57	1.98	1.60–2.44

^1^Adjusted for women age, education, number of children, place of residence and wealth quintile.

Women with high autonomy in the domain of attitude to violence (i.e. strong opposed the notion of violence) are 1.66 times more likely to receive at least seven essential ANC components as compared with women who ranked low on attitude to violence (i.e. least strongly opposed the notion of violence). Women who enjoy medium (aOR = 1.66) or high (aOR = 2.78) social independence had higher odds of receiving quality ANC consultations in contrast with low socially independent women. Women who had medium (aOR = 1.34) or high autonomy (aOR = 1.98) in household decision making were more likely to receive quality ANC consultations as opposed to women who possess low autonomy in household decision making.

## Discussion

This pioneering study examined the relationship between women empowerment and quality of antenatal care in Pakistan. The results of this study directly feed into the SDGs 3 and 5 which focuses on interlinked issues of health and gender inequalities.

### Women empowerment

About an equal proportion of the women enjoy low, medium and high level of social independence which is the preconditions that enable women to achieve their desired goals. Given the parameters used in this domain, low and medium level of social independence in Pakistan may be attributed to low female education, early age marriages, and a trend among men to marry a younger woman [25]. These preconditions, combined with other socio-cultural factors, are responsible for their low instrumental agency—which refers to women’s ability to make decisions about household affairs, including going outside of home [26]. In rural and traditional families in Pakistan, women are usually not allowed to go alone to seek health care. They remain dependent on their husband, or elder family members for going outside of home to visit relatives, shopping and seeking health care [27].

Although relatively low, but one-third of the women fell in the low empowerment category in the domain of attitude towards violence. This means that they tend to accept the notion of wife beating by husbands to a certain extent. Socio-cultural norms shape women intrinsic agency to accept male dominancy and justify violence against them in certain circumstances. At times, such behaviour is also justified on religious grounds [28]. It is consistent with a study of Pakistan which shows that intimate partner violence is considered as a private matter and usually a justifiable response to misbehaviour on the part of the wife [29, 30]. Interestingly men are less likely to justify wife beating as compared to women in Pakistan [24] and women are considered wise if they remain obedient in tough situation particularly during inter-familial conflicts in rural areas [31].

### Quality of antenatal care

Like other LMICs [11], our study also found a substantive difference between crude coverage and quality coverage of ANC. A possible reason for low quality coverage in Pakistan could be delayed seeking of ANC which probably contributes missing the opportunity to not receiving all essential components of ANC care [32]. More specifically, we found that only 20% women consumed iron tablets/syrup relative to utilisation of other essential ANC components (see Fig 2) which may be attributed to the non-availability of supplements, forgot to take, family members do not allow to consume and women consider them as contraceptive [33, 34]. Overall, there is a critical gap in the quality of ANC in our health-care system that requires urgent interventions and implementation of the WHO new guidelines on antenatal care to improve the quality of health-care services [3].

### Women empowerment and quality of ANC

Our study revealed that all three measures of empowerment had a positive effect on utilisation of quality antenatal care. It is evident that more empowered women are more likely to receive quality care during pregnancy in Pakistan. This relationship was incremental in most cases (p-value of trend was <0.05) which indicates that the odds of utilisation linearly increase with the increase in level of empowerment. Our study findings are consistent with the result of a systematic review on women’s empowerment and access to care and health status for mothers and their children from LMICs [35].

With regard to the dimension of decision making, our measure included items of women’s mobility, health care, and major household purchases. Women’s instrumental agency gives them control over reproductive and sexual decisions [36]. For example, autonomy for mobility increases women’s freedom to visit food markets and attend health centre appointments for themselves and for their children along with visiting friends and relatives. As a result, women acquire resources such as information and support [37] which help to improve maternal and child health care [38].

Women who are more socially independent, may be more likely to recognize the value of formal health services and have the skills and resources to opt for these services. It may be possible that women with more equitable gender norms are better able to advocate for themselves during ANC and thus actually receive better services. With respect to utilisation of essential ANC components, socially independent women are able to express themselves to health care providers as being worthy of care, in turn service providers provide better health services to them.

The domain of women’s attitude towards violence also showed a significant relationship with quality ANC. This finding was consistent with another study in Albania where women’s attitudes towards domestic violence were associated with antenatal care and postnatal care utilization [39]. Research suggests that social norms around violence against women are directly relate to how women are treated in health facilities [40, 41]. Acceptability of violence against women at the community level may trickle down to how women are provided services in facilities by service providers and how health systems support women in general. On the contrary, women who report less acceptance of domestic violence may be living in households and communities that also support such views and improve women’s quality of care in general. Women who held more progressive views in the sexual relationship domain may reflect more egalitarian relationships with their husbands. It can be hypothesized that women who are more empowered in regard to sexual relationships with their husbands may also have increased support from husbands in receiving quality of ANC services. Evidence show that economic status of household strongly influences the health seeking behaviour of women for maternal and child health. For future research, it will be interesting to study whether women’s level of empowerment play a moderating role in minimizing poor-rich inequalities for range of maternal-health seeking behaviours.

### Strengths and limitations

Strengths of our study include use of large-scale national data and use of SWPER–a validated multi-dimensional measure of women’s empowerment for LMICs. In terms of the limitation, we excluded samples of AJK and Gilgit Baltistan due to the use of different sampling design. It is however important to note that the excluded regions contribute minimally to country’s population. The cross-sectional nature of data does not enable us to infer causal inferences between exposure and outcomes. Our measures of women’s empowerment solely based on women’s report, whereas other studies find it discrepant with husband’s report of women’s empowerment than their wives [42]. Lastly, the DHS collected data from women who gave birth in previous five years, hence there is the possibility of recall bias due to self-reported answers could be a limitation in this study.

## Conclusions

The quality of ANC coverage and quality ANC consultation are considerably low in Pakistan. This study highlights the gaps in seeking quality of ANC care utilization during pregnancy. Furthermore, our analysis shows that more than one-third of women had low empowerment in each domain that reflects gender inequality at the household level. Women’s empowerment related to social independence, gendered beliefs about violence, and decision-making–each has an independent positive effect on utilisation of quality antenatal care. The study results suggest that each domain of women empowerment is significantly associated with seeking quality of ANC coverage and ANC consultation. More empowered women in each domain seek quality of ANC care in Pakistan. Thus, efforts directed towards empowering women could be effective strategy to improve utilisation of quality antenatal care in Pakistan. Empowering women can be helpful to achieve sustainable development goals 3 and 5 to improve health services utilization and achieve gender equality.
